# An *ANK1* IVS3-2A>C mutation causes exon 4 skipping in two patients from a Chinese family with hereditary spherocytosis

**DOI:** 10.18632/oncotarget.22936

**Published:** 2017-12-05

**Authors:** Xiong Wang, Liyan Mao, Na Shen, Jing Peng, Yaowu Zhu, Qun Hu, Yanjun Lu

**Affiliations:** ^1^ Department of Laboratory Medicine, Tongji Hospital, Tongji Medical College, Huazhong University of Science and Technology, Wuhan, China; ^2^ Department of Pediatrics, Tongji Hospital, Tongji Medical College, Huazhong University of Science and Technology, Wuhan, China

**Keywords:** hereditary spherocytosis, anemia, ANK1, splenectomy

## Abstract

Hereditary spherocytosis (HS) is a congenital hemolytic anemia that affects the cell membrane of red blood cells and is characterized by the presence of spherical-shaped erythrocytes in the peripheral blood film. The clinical manifestation of HS ranges from asymptomatic to severe cases that require transfusion during early childhood. HS is caused by mutations in red blood cell membrane protein encoding genes, including ANK1, EPB42, SLC4A1, SPTA1, and SPTB. Mutations of the ANK1 gene account for 75% of all HS cases, and these particular mutations are typically inherited in an autosomal dominant manner. In this study, heterozygous an ANK1 IVS3-2A>C mutation was identified in a 7-year-old girl with Coombs-negative and severe hemolytic jaundice using targeted next-generation sequencing (NGS) and Sanger sequencing. Spherocytes were observed in a peripheral smear. Osmotic fragility was increased, and glucose-6-phosphate dehydrogenase (G6PD) activity was normal. A genetic mutation screen for α- and β-thalassemia was negative. Autoimmune antibody tests were negative. Both the girl and her affected father received a splenectomy. Patient-derived peripheral blood mononuclear cells showed skipping of exon 4 in the mRNA, which confirmed the splicing mutation effect of the ANK1 IVS3-2A>C mutation. Moreover, the anemia was ameliorated after splenectomy. Our results demonstrate that the ANK1 IVS3-2A>C mutation may lead to exon 4 skipping of the ANK1 gene and cause HS.

## INTRODUCTION

Hereditary spherocytosis (HS) is a common form of inherited hemolytic anemia that has a prevalence ranging from 1:2,000 to 1:5,000 in Caucasians and 1:100,000 in Chinese individuals [1, 2]. It is caused by defects in red blood cell membrane proteins, such as ankyrin, protein 4.2, band 3 protein, α-spectrin, and β-spectrin, which are encoded by the ANK1, EPB42, SLC4A1, SPTA1, and SPTB genes, respectively [3]. The main clinical manifestations of HS are highly variable, including signs of anemia, jaundice, gallstones, and splenomegaly [4]. Approximate 75% of all HS cases are inherited in an autosomal dominant manner. Management of HS patients may include blood transfusions and splenectomy to reduce hemolysis and clinical symptoms [3].

The ANK1 gene is located on chromosome 8p11.1 and encodes several alternatively spliced isoforms [5]. Mutations in the ANK1 gene are responsible for the majority of all HS cases, followed by mutations in the SLC4A1 and SPTB genes. Ankyrin consists of a multiple ankyrin repeat N-terminal domain, a spectrin-binding center region, and a regulatory C-terminal domain. The major function of ankyrin is to stabilize the membrane structure by interacting with spectrin, protein 4.2, and band 3 protein. Ankyrin provides a high-affinity linkage between the spectrin-actin based membrane skeleton and the red blood cell membrane [6]. Next-generation sequencing (NGS) has been widely used to perform genetic diagnosis of hemolytic anemia, including HS [7].

In this study, a heterozygous ANK1 IVS3-2A>C mutation was identified by NGS and Sanger sequencing in two patients from a Chinese family, both of whom received a splenectomy. Patient-derived peripheral blood mononuclear cells showed skipping of exon 4 in the mRNA.

## RESULTS

### Clinical features of a Chinese family with HS

**Figure 1:**
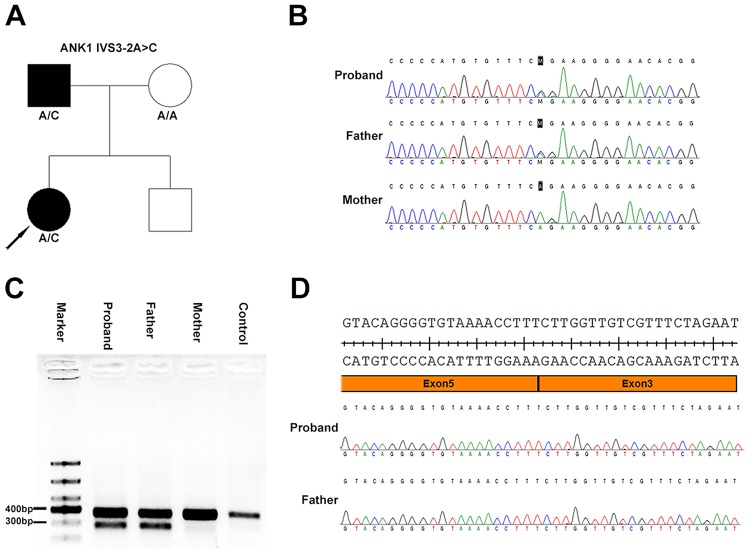
Heterozygous ANK1 IVS3-2A>C mutation in two patients from a Chinese family with hereditary spherocytosis **A**. Family tree and the genotype at the ANK1 IVS3-2 position. Squares and circles denote male and female, respectively. Black symbols denote patients with HS. **B**. Sanger sequencing identified an ANK1 IVS3-2A>C mutation. **C**. DNA gel of the PCR products spanning exon 2 to 6 from cDNA transcribed from mRNA isolated from patient-derived peripheral blood mononuclear cells. Both the girl’s mother and a healthy control yielded a normal 381 bp band, while the girl and her father produced two bands, a normal band and a smaller band. **D**. Sanger sequencing of the smaller band separated in panel C. These results showed a complete deletion of exon 4 of the ANK1 gene.

A 7-year-old girl with anemia, jaundice, and splenomegaly was diagnosed with HS when she was 1 year of age according to her clinical symptoms, laboratory tests, and a positive family history. Osmotic fragility was increased, and G6PD activity was normal. A genetic mutation screen for α- and β-thalassemia was negative. Autoimmune antibody tests were negative. Spherical-shaped erythrocytes were found in the peripheral blood film. She recently received a splenectomy due to severe anemia, and the anemia was ameliorated after the splenectomy. Her affected father was diagnosed with HS when he was young and received a splenectomy when he was 10 years old. The family tree is shown in Figure 1A, and the laboratory tests are summarized in Table 1.

**Table 1:** Laboratory test results

Test	Result	Reference
RBC	2.58 × 1012/L (↓)	4.3-5.8 × 1012/L
MCV	93 fL	82-100 fL
MCH	29.5 pg	27-34 pg
MCHC	31.7 g/dL	31.6-35.4 g/dL
TBIL	118.1 μmol/L (↑)	3.4-20.5 μmol/L
IBIL	112.1 μmol/L (↑)	≤ 13.3 μmol/L
BRD	6.0 μmol/L (↑)	0-6.8 μmol/L

RBC: red blood cell, MCV: mean corpuscular volume, MCH: mean corpuscular hemoglobin, MCHC: mean corpuscular hemoglobin concentration, TSB: total bilirubin, IBIL: indirect bilirubin, BRD: bilirubin direct.

### NGS output and coverage

All coding exons and adjacent intronic regions of HS-related genes were sequenced on the Ion torrent PGM, and achieved an average output of 822,775 mapped reads and 98.65% on-target specificity. The mean depth was 2,282 folds. All target bases were covered at least once, 97.11% were covered at least 100 times, and 93.49% were covered at least 500 times. The mean uniformity of base coverage is 94.04%.

### Mutation detection and Sanger sequencing validation

NGS identified a total of 56 variants subjected to a process to select pathogenic mutation using the filtration process and annotation. A heterozygous ANK1 IVS3-2A>C mutation was identified.

Sanger sequencing of the ANK1 IVS3-2A>C mutation was performed on all family members. The results showed that both the patient and her father carried this mutation, while her mother did not (Figure 1B). The genetic analysis for her brother was not available.

Moreover, the ANK1 IVS3-2A>C mutation (hg19: chr8:41585526A>C) was not found in the 1000G, ExAC, or HGMD databases.

Amplification of the sequence spanning exon 2-6 of the ANK1 mRNA reversely transcribed cDNA produced only one band in her mother and a healthy control, while the patient and her father produced a normal band and a smaller band separated by DNA agarose gel (Figure 1C). Sequencing of the smaller bands showed skipping of exon 4 in the mRNA (Figure 1D), which led to a direct junction of exon 3 and exon 5. These results confirm the splicing mutation effect of the ANK1 IVS3-2A>C mutation.

### Computational analysis

The effect of the ANK1 IVS3-2A>C mutation was computationally analyzed using two splice site prediction programs, GENIE and MES, and the result is summarized in Table 2. Both algorithms predicted a weakening of the 3’ splice-site consensus sequence of exon 4.

**Table 2:** Bioinformatics prediction of splicing mutations by GENIE and MES

Program	Wild type	Mutant
GENIE	4.3	-6.6
MES	7.69	-0.35

### Genetic and phenotype association analysis

Genetic tests showed a heterozygous ANK1 IVS3-2A>C mutation in this family, and both patients carried this mutation. Jaundice and splenomegaly were observed in the patients. Consistent with the genetic findings, anemia was ameliorated soon after splenectomy.

## DISCUSSION

ANK1 mutations are responsible for the majority of all cases of HS. Patients carrying ANK1 mutations mainly exhibit an autosomal dominant pattern of inheritance, but some of these mutations are inherited in an autosomal recessive pattern [8, 9]. These mutations cover the entire ANK1 gene, including the promoter region, and de novo mutations occur with high frequency [10]. To date, a total of 59 mutations are included in the HGMD public database. Small deletion and nonsense mutations account for more than 50% of all mutations in the ANK1 gene. Seven splicing mutations, occurring in IVS1, 16, 20, 22, 28, and 38, are included in the HGMD database. In this study, patients displayed an autosomal dominant pattern of inheritance. Both the girl and her affected father carried a heterozygous ANK1 IVS3-2A>C mutation identified by NGS and confirmed by Sanger sequencing.

NGS has been widely used for genetic diagnosis of hereditary red blood cell membrane disorders, including HS [11]. In this study, the mean depth was 2,282 folds, and 93.49% target bases were covered at least 500 times. Target bases covered fewer than 20 times and the uncovered regions were analyzed using Sanger sequencing. These results confirmed the presence of the variants identified by NGS.

The major form of Ankyrin includes an 89-kd N-terminal region composed of 24 conserved repeats, a membrane domain containing binding site for band 3, a 62-kd central domain containing binding sites for spectrin and vimentin, and a 55-kd C-terminal regulatory domain [12]. In this family, a ANK1 IVS3-2A>C mutation occurred in the N-terminus region. Sequencing of cDNA transcribed from mRNA isolated from patient-derived peripheral blood mononuclear cells revealed that the ANK1 IVS3-2A>C mutation resulted in the skipping of exon 4 in the ANK1 gene. Exon 4 deletion did not affect the reading frame of the ANK1 gene, but it may produce a mutant protein that lacks the 33 amino acids encoded by exon 4. The ANK1 IVS3-2A>C mutation may disrupt the binding of band 3 protein. In silico splicing prediction programs also revealed that the ANK1 IVS3-2A>C mutation weakened the 3’ splice-site consensus sequence of exon 4.

Splenectomy is therapeutic for patients with HS after the age of 6 [13, 14]. After surgery, the anemia and jaundice of the 7-year-old girl were ameliorated within one week, although increased platelet counts due to the splenomegaly continued for two weeks. Her father, who received a splenectomy when he was young, is now asymptomatic.

In summary, our results demonstrate that an ANK1 IVS3-2A>C mutation may lead to exon 4 skipping of the ANK1 gene and may be responsible for HS in two patients from a Chinese family.

## MATERIALS AND METHODS

### Subjects

Samples from a 7-year-old girl suspected of HS and her parents were collected. The girl received a splenectomy due to severe anemia. Her father also received this surgery when he was 10 years old. Osmotic fragility was increased, and G6PD activity was normal. Genetic mutation screens for α- and β-thalassemia was negative. Autoimmune antibody tests were negative. Spherical-shaped erythrocytes were found in the peripheral blood film. Written informed consent was obtained from all subjects, and this work was formally approved by the Ethics Committee of Tongji Hospital, Tongji Medical College, Huazhong University of Science and Technology. All procedures were performed in accordance with the approved guidelines.

### Ampliseq NGS panel design

A custom NGS panel covering all exons and adjacent introns of the ANK1, SLC4A1, SPTB, SPTA1, and EPB42 genes was designed using the Ampliseq tool (http://www.ampliseq.com) with coverage of 96.71%, 96.53%, 99.54%, 100%, and 100%, respectively. The uncovered sequences were amplified and sequenced by Sanger sequencing.

### NGS

DNA libraries were built according to the Ampliseq™ Library Preparation Kit 2.0 using 10 ng of input DNA from PBMC in two primer-pools with 118 and 115 amplicons. The PCR products of the two pools were mixed together and purified using AMPure XP beads (Beckman Coulter, Brea, CA, USA) and quantified using the Ion Library TaqMan™ Quantitation Kit. Prepared libraries were pooled in equal amounts and diluted to a concentration of 100 pM. Samples (2 μL) of the pooled library was further enriched using a One-Touch machine and ES Instrument using the Ion PGM™ Hi-Q™ OT2 Kit. Enriched products were sequenced using the Ion Torrent PGM Hi-Q Sequencing Kit on PGM using the Ion 316™ chip.

### Bioinformatic analysis

Trimmed raw data were aligned to the hg19 human reference genome. Coverage and variant identification analysis was performed on the Ion Torrent Server 4.4.2. Variants were annotated with the Ion Reporter 5.0 software according to the nomenclature recommended by the Human Genome Variation Society (HGVS, http://www.hgvs.org). All identified variants and sequences with less than 20 × coverage depth were visually verified using the Integrative Genomics Viewer 2.3.8 (Broad Institute, Cambridge, MA, USA). Clinical significance of selected variants was made according to the 1000G (http://asia.ensembl.org/index.html), ExAC (http://exac.broadinstitute.org/), and HGMD (http://www.hgmd.cf.ac.uk/ac/index.php) databases.

### Sanger sequencing

Sanger sequencing was performed as previously described. Briefly, genomic DNA was extracted from PBMCs using the QIAamp DNA blood mini kit. Coding exons and splice junctions of HS-related genes were amplified for selected mutations, failed amplified or uncovered regions. RNA was extracted from PBMCs using Trizol and reversely transcribed into cDNA for sequencing. Sanger sequencing was performed bi-directionally on an ABI 3500 Dx. NM_001142446.1 was used as a reference transcript for the ANK1 gene. Primers used to amplify the region spanning exon 2 to 6 of the ANK1 gene from cDNA are as follows: ANK1 E2-6 RT F (381 bp), 5’-CAGAATGGGTTGAATGGCT-3’; ANK1 E2-6 RT R (381 bp), 5’-CTTGGTGCCGTAGTTGATG-3’. Primers used to amplify exon 4 from DNA are as follows: ANK1 E4 F (545 bp), 5’-GTCATAGAGAACAGGCTGGATC-3’; ANK1 E4 R (545 bp), 5’-GGCTTAGGTCCAAAACAATTC-3’.

### Splicing predictions

To assess the presumptive effects of the splicing mutation, two in silico prediction tools were applied: the GENIE program (http://rulai.cshl.edu/new_alt_exon_db2/HTML/score.html) and MES using the Weight Matrix Model (http://genes.mit.edu/burgelab/maxent/Xmaxentscan_scoreseq_acc.html) [15]. The high value indicates a high possibility of being a splicing site.
